# Predictors and prevention of flow insufficiency due to limited flow demand

**DOI:** 10.1186/s13019-014-0188-3

**Published:** 2014-12-04

**Authors:** Hiroyuki Nakajima, Atsushi Iguchi, Mimiko Tabata, Hiroyuki Koike, Kozo Morita, Ken Takahashi, Toshihisa Asakura, Shigeyuki Nishimura, Hiroshi Niinami

**Affiliations:** The Department of Cardiovascular Surgery, Saitama International Medical Center, Saitama Medical University, 1397-1 Yamane, Hidaka 350-1298, Saitama, Japan; The Department of Cardiology, Saitama International Medical Center, Saitama Medical University, 1397-1 Yamane, Hidaka 350-1298, Saitama, Japan

**Keywords:** Coronary artery bypass graft, CABG arterial grafts, CABG venous grafts, Myocardial ischemia, Off-pump surgery

## Abstract

**Background:**

We investigated the impacts of flow demand and native coronary stenosis on graft flow and patency.

**Methods:**

We reviewed the angiograms of 549 bypass grafts in 301 patients who underwent off-pump coronary artery bypass grafting since 2007. Grafts consisted of 237 internal thoracic artery to left anterior descending artery; 97 internal thoracic artery and 52 saphenous vein grafts to left circumflex artery; and 109 gastroepiploic artery and 54 saphenous vein grafts to right coronary artery. We selected only individual bypass grafts created as the sole bypass graft to the coronary vascular region. Flow insufficiency was defined as ≤ 20 ml/min measured intraoperatively. When a significant difference in the incidence of flow insufficiency or “not functional” occurred between higher and lower values rather than the particular minimal luminal diameter value, the highest value was defined as the cut-off minimal luminal diameter. Distal lesions were defined as stenosis at segment #4, 7, 8, 12, 13, 14, or 15.

**Results:**

Flow insufficiency was found in 112/549 (20.4%) bypass grafts. For internal thoracic artery to left circumflex artery grafts, the cut-off minimal luminal diameter for proximal and distal lesions was 1.25 mm and 0.75 mm, respectively. For gastroepiploic artery to right coronary artery grafts, the cut-off minimal luminal diameter was 0.82 mm for proximal lesions (p = 0.005), while 10 (71%) of 14 gastroepiploic artery grafts for distal lesions presented with flow insufficiency. Univariate and multivariate analysis identified a distal lesion (odds ratio (OR): 3.12, p < 0.0001); minimal luminal diameter greater than the cut-off value (OR: 3.64, p < 0.0001); right coronary artery (OR: 18.2, p = 0.0002) and left circumflex artery (OR; 2.29, p = 0.009) grafting; and a history of myocardial infarction in the grafted region (OR: 2.21, p = 0.02) as significant predictors of flow insufficiency.

**Conclusions:**

Both competitive flow and insufficient flow demand cause graft failure. For distal lesions, more severe stenosis is necessary to avoid graft failure, compared with proximal lesions. A revascularization strategy for distal lesions should be discussed separately from that for proximal lesions.

**Electronic supplementary material:**

The online version of this article (doi:10.1186/s13019-014-0188-3) contains supplementary material, which is available to authorized users.

## Background

Following coronary artery bypass grafting (CABG), graft flow is determined by flow demand in the grafted region balanced against the native coronary flow. Prevention of competitive flow has been discussed for appropriate graft flow and long-term patency of arterial grafts. However, the impacts of flow demand in the revascularized region on graft flow and patency have not been fully delineated.

Insufficient graft flow in the arterial graft is a reported major predictor of graft failure within one year [1],[2]. Thrombosis and intimal hyperplasia can cause failure of saphenous vein grafts (SVG) within 1 year, while atherosclerosis is considered the main cause of SVG failure after 1 year [3].

In this study, we focused on prevention of graft failure relatively early postoperatively. We examined the possible factors associated with flow demand, which included the stenosis location, history of myocardial infarction in the grafted region, ventricular function, and native coronary flow, which included the cut-off minimal luminal diameter (MLD) combined with the stenosis location.

## Methods

We retrospectively reviewed the pre- and postoperative coronary angiograms and flowmetry results of 301 patients who underwent off-pump CABG and postoperative angiography between 2007 and June 2013. To examine the size of the revascularized region, we selected only simple configuration bypass grafts for analysis. We included 549 individual bypass grafts (Table 1), that were created as the only bypass graft to the coronary vascular region, such as the left anterior descending artery (LAD), left circumflex artery (LCX), and right coronary artery (RCA), and that were evaluated by postoperative catheter angiography. The study was approved by Institutional Review Board of Saitama International Medical Center. Grafts included 237 in-situ internal thoracic artery (ITA) to LAD bypass grafts; 97 in-situ ITA to LCX; 52 aorto-coronary SVG to LCX; 109 in-situ gastroepiploic artery (GEA) to RCA; and 54 aorto-coronary SVG to RCA (Table 2). We excluded sequential and composite grafts and bypass grafts with other combinations of graft material and target branch, and patients who had no postoperative angiography. We also excluded bypass grafts which were anastomosed to two or more targets in the same vascular region. For example, when there was a bypass graft to the diagonal branch, the concomitant in-situ ITA to LAD bypass graft was excluded. One in-situ ITA that had localized dissection because of surgical trauma was also excluded. There were 237 males and 64 females in this study with a mean age of 67 ± 9 years. Of these, 120 (52%) patients had diabetes mellitus. The number of distal anastomoses was 3.2 ± 1.1 per patient and 20 (6.6%) patients had persistent atrial fibrillation before and during operation.

**Table 1:** **Patients’ baseline characteristics**

No. of patients	301
Age (y)	67 ± 9
Male/Female	237/64
Hypertension	193 (64%)
Hyperlipidemia	174 (58%)
Diabetes	152 (50%)
Atrial Fibrillation	20 (7%)
Intraaortic balloon pump	51 (17%)
LV mass < 250 g	67 (22%)
Ejection fraction of LV (%)	56 ± 18
Ejection fraction of LV < 40%	45 (15%)
Total distal anastomoses	974
Targets per patient	3.2 ± 1.0

LV: left ventricle.

**Table 2:** **Bypass graft characteristics**

Bypass grafts analyzed	549
in-situ ITA to LAD	237
in-situ ITA to LCX	97
aorto-coronary SVG to LCX	52
in-situ GEA to RCA	109
aorto-coronary SVG to RCA	54
History of MI in the grafted region	88 (16%)
History of PCI in the grafted region	73 (13%)

GEA: gastroepiploic artery, ITA: internal thoracic artery, LAD: left anterior descending artery, LCX: left circumflex artery, MI: myocardial infarction, PCI: percutaneous coronary intervention, RCA: right coronary artery, SVG: saphenous vein graft.

Our standard operative technique is off-pump CABG with in-situ arterial grafts. All grafts were ≥ 1.5 mm or more in diameter. The bilateral ITA was used for patients with an active lifestyle who were < 80 years old. For patients with severe chronic obstructive pulmonary disease, chronic kidney disease, or insulin-dependent diabetes mellitus, the SVG was commonly used for LCX grafting. For RCA grafting, the use of GEA depended on the surgeon’s decision, based on estimated operative risk, severity of the proximal stenosis, vessel size, or a previous or future abdominal operation. After completion of anastomosis, graft flow was measured by a transit time flow meter (Medi-Stim AS, Oslo, Norway) and recorded for all patients. To determine the influence of flow demand, we examined the grafted vascular region (such as LAD, LCX, or RCA), gender, history of myocardial infarction in the grafted region and any history of percutaneous coronary intervention to the grafted region, left ventricular ejection fraction, atrial fibrillation, and left ventricular mass.

Early postoperative coronary angiography was performed during hospitalization approximately two weeks after surgery. Cardiologists independently evaluated the severity of native coronary artery stenosis and anatomical and functional graft patency. The definition MLD in the present study was as follows: MLD (mm) was measured at the narrowest stenotic lesion proximal to the anastomotic site in preoperative or early postoperative angiography using the Toshiba Cardio Agent® system (Toshiba Medical Systems Co., Tokyo, Japan).

Proximal lesions were defined as stenosis at segment #1, 2, 3, 5, 6, or 11, while distal lesions were defined as stenosis at segment #4, 7, 8, 12, 13, 14, or 15. Flow insufficiency (FI) was defined as ≤ 20 ml/min during intraoperative transit-time flowmetry. Not functional (NF) was defined as occlusion and string sign, which was a diffuse narrowing of the arterial conduit opacified with delayed antegrade flow. When a significant difference in the incidence of FI or NF was detected between higher and lower values rather than the particular MLD value, the highest value was defined as the cut-off MLD.

The mean follow-up period was 12 ± 15 months. Late angiography (> 6 months) was performed for 23 (7.6%) patients for clinical reasons and the mean interval between CABG and angiography was 2.5 ± 7.5 (0.1–46) months.

### Statistical analysis

The continuous variables are expressed as means ± standard deviation. The data of two independent groups were compared by chi-square test and multivariate analysis was performed using logistic regression. A cut-off p-value of 0.20 in the univariate analyses was used to select variables for inclusion in multivariate models. Statistical analyses were performed using SPSS software (SPSS8.0 Inc., Chicago, IL, USA) and outcome differences were considered statistically significant with p < 0.05.

## Results

**Figure 1:**
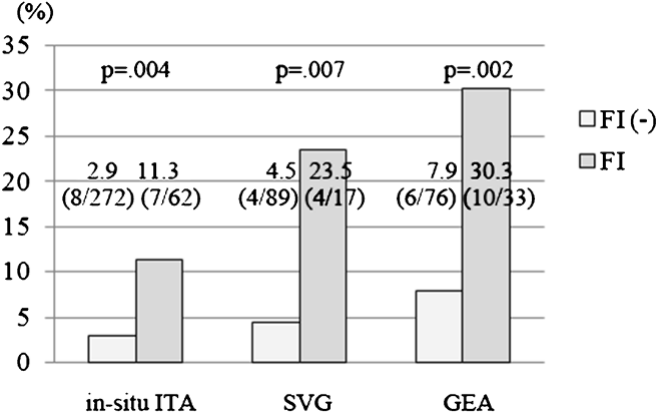
**Incidence of not functional (NF) for in-situ ITA, SVG, and GEA with and without flow insufficiency (FI).** The rate of NF was significantly higher in bypass grafts with FI for all graft materials. FI: flow insufficiency, GEA: gastroepiploic artery, ITA: internal thoracic artery, NF: not functional, SVG: saphenous vein graft.

**Figure 2:**
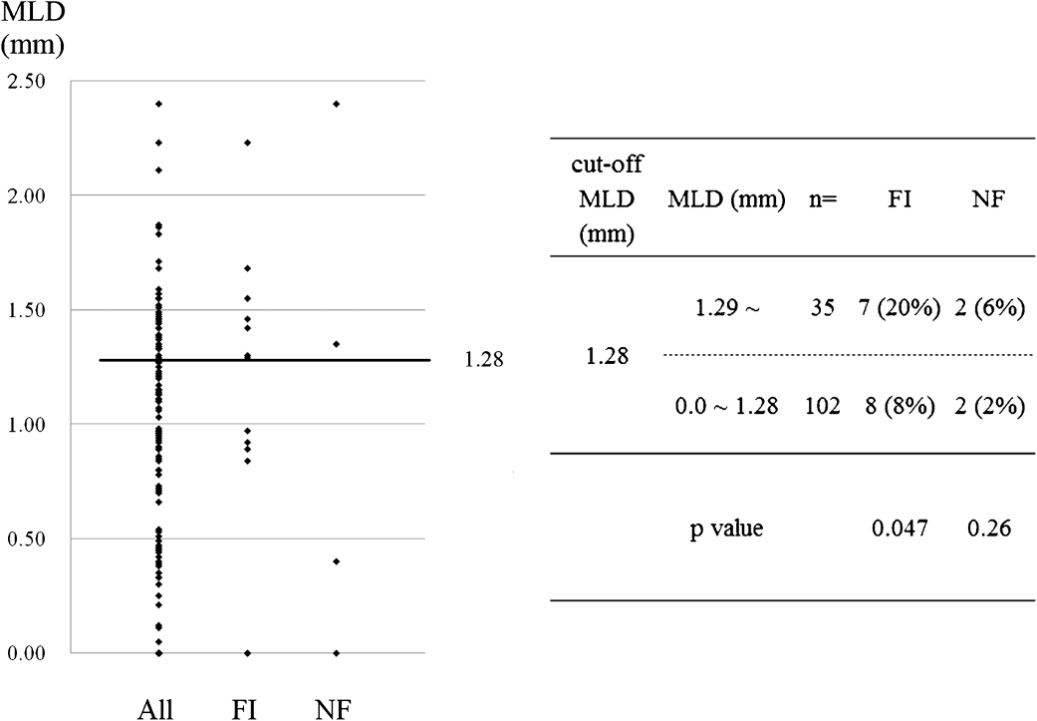
**Minimal luminal diameter (MLD) with and without flow insufficiency (FI) and not functional (NF) in in-situ ITA to LAD with proximal lesions.** The incidence of FI was significantly higher in the targets with MLD > 1.28, which was defined as the cut-off MLD. FI: flow insufficiency, ITA: internal thoracic artery, MLD: minimal luminal diameter, NF: not functional.

As shown in Figure 1, there were significant correlations between FI and NF for GEA (p = 0.002), SVG (p = 0.0007), and ITA (p = 0.004) (Figure 1). Overall, 112/549 (20.4%) bypass grafts were assessed as FI and 39/549 (7.0%) were assessed as NF and the incidences of FI and NF are summarized in Table 3. For ITA to LAD, the cut-off MLD was 1.28 mm for proximal lesions (p = 0.04) (Figure 2) and 0.77 mm for distal lesions (p = 0.004). For ITA to LCX, the cut-off MLD was 1.25 mm for proximal lesions (p = 0.002), and 0.75 mm for distal lesions (p = 0.005). There was no significant cut-off MLD for SVG to LCX. For GEA to RCA, the cut-off MLD was 0.82 mm for proximal lesions (p = 0.005), while 10 of 14 (71%) GEA for distal lesions presented with FI, irrespective of MLD. For SVG to RCA the cut-off MLD was 1.29 mm for proximal lesions (p = .001), while 8 of 17 (47%) SVG for distal lesions presented with FI with no cut-off value (Table 3).

**Table 3:** **Cut-off MLD and the incidence of LF and NF**

Graft	Target	Location of stenosis	n	LF	NF	Cut-off MLD (mm)	MLD (mm)	n	LF	NF
in-situ ITA	LAD	Proximal	137	15 (11%)	4 (3%)	1.28	1.29~	35	7 (20%)	2 (6%)
							0.0 ~ 1.28	102	8 (8%)	2 (2%)
							p-value*		0.047	0.26
		Distal	100	18 (18%)	5 (5%)	0.77	0.78~	42	13 (31%)	4 (10%)
							0.0 ~ 0.77	58	5 (9%)	1 (2%)
		p-value (vs. proximal)		0.12	0.41		p-value*		0.004	0.002
in-situ ITA	LCX	Proximal	48	8 (17%)	3 (6%)	1.25	1.26~	19	7 (37%)	2 (11%)
							0.0 ~ 1.25	29	1 (3%)	1 (3%)
							p value*		0.002	0.32
		Distal	49	21 (43%)	3 (6%)	0.75	0.76~	26	16 (62%)	3 (12%)
							0.0 ~ 0.75	23	5 (22%)	0
		p-value (vs. proximal)		0.009	0.98		p-value*		0.005	0.09
SVG	LCX	Proximal	24	1 (4%)	0	-				
		Distal	28	2 (7%)	3 (11%)	-				
		p-value (vs. proximal)		0.65	0.10					
in-situ GEA	RCA	Proximal	95	23 (24%)	13 (14%)	0.82	0.83~	22	8 (36%)	7 (32%)
							0.0 ~ 0.82	73	15 (21%)	6 (8%)
							p-value*		0.13	0.005
		Distal	14	10 (71%)	3 (21%)	-				
		p-value (vs. proximal)		0.0003	0.44					
SVG	RCA	Proximal	37	6 (16%)	2 (5%)	1.29	1.30~	11	4 (36%)	2 (18%)
							0.0 ~ 1.29	26	2 (8%)	0
							p-value*		0.03	0.02
		Distal	17	8 (47%)	3 (18%)	-				
		p-value (vs. proximal)		0.02	0.15					

FI: flow insufficiency, GEA: gastroepiploic artery, ITA: internal thoracic artery, LAD: left anterior descending artery, LCX: left circumflex artery, MLD: minimal luminal diameter, NF: not functional, RCA: right coronary artery, p-value*: larger vs. smaller than the cut-off value.

There were significant differences in the incidence of FI between proximal and distal lesions for ITA to LCX (p = 0.009), GEA to RCA (p = 0.0003), and SVG to RCA (p = 0.02) (Table 3). There was no cut-off value for SVG to LCX with proximal and distal lesions, and SVG and GEA to RCA with distal lesions.

Univariate logistic regression analyses demonstrated that renal failure, ejection fraction < 40%, a distal lesion, graft type, MLD > cut-off value, grafted region of LCX and RCA, and a history of myocardial infarction in the grafted region significantly correlated with FI. Using multivariate analysis, MLD greater than the cut-off value (odds ratio (OR): 3.64, p < 0.0001); a distal lesion (OR: 3.12, p < 0.0001); RCA (OR: 18.2, p = 0.0002) and LCX (OR: 2.29, p = 0.009) regions; and a history of myocardial infarction in the grafted region (OR: 2.21, p = 0.02) were significant predictors of FI (Table 4).

**Table 4:** **Predictors of flow insufficiency for the 549 bypass grafts**

Variables	Odds ratio	95% CI	p-value
*Univariate analysis*			
Hyperlipidemia	0.92	(0.61–1.40)	0.70
Hypertension	1.13	(0.73–1.74)	0.59
Diabetes	1.01	(0.67–1.53)	0.96
Renal failure	0.20	(0.048–0.85)	0.03
Ejection Fraction < 40%	0.42	(0.21–0.84)	0.01
LV mass < 250 (g)	1.12	(0.68–1.83)	0.66
Intraaortic balloon pump	0.66	(0.36–1.21)	0.18
Atrial Fibrillation	0.98	(0.32–2.98)	0.96
Distal lesion (vs. proximal lesion)	1.78	(1.17–2.70)	0.007
Graft; in-situ GEA (vs. ITA)	1.91	(1.16–3.12)	0.01
Graft; SVG (vs. ITA)	0.84	(0.47–1.51)	0.55
MLD > cut-off value	3.25	(2.11–5.01)	<0.0001
Female (vs. male)	1.46	(0.91–2.34)	0.12
Grafted region; LCX (vs. LAD)	1.69	(0.99–2.89)	0.055
Grafted region; RCA (vs. LAD)	2.51	(1.52–4.13)	0.0003
History of PCI in the grafted region	1.58	(0.90–2.76)	0.11
History of MI in the grafted region	1.96	(1.17–3.26)	0.009
*Multivariate analysis*			
Renal failure	0.24	(0.055–1.13)	0.07
Ejection Fraction < 40%	0.47	(0.22–1.01)	0.052
Intraaortic balloon pump	0.85	(0.42–1.69)	0.64
Distal lesion (vs. proximal lesion)	3.12	(1.86–5.22)	<0.0001
Graft; in-situ GEA (vs. ITA)	0.27	(0.058–1.24)	0.09
Graft; SVG (vs. ITA)	0.19	(0.052–0.72)	0.01
MLD > cut-off value	3.64	(2.20–6.03)	<0.0001
Female (vs. male)	1.56	(0.92–2.64)	0.10
Grafted region; LCX (vs. LAD)	2.29	(1.22–4.27)	0.009
Grafted region; RCA (vs. LAD)	18.21	(3.95–84.02)	0.0002
History of PCI in the grafted region	1.23	(0.61–2.49)	0.56
History of MI in the grafted region	2.21	(1.13–4.31)	0.02

GEA: gastroepiploic artery, ITA: internal thoracic artery, LAD: left anterior descending artery, LCX: left circumflex artery, MI: myocardial infarction, MLD: minimal luminal diameter, PCI: percutaneous coronary intervention, RCA: right coronary artery.

## Discussion

Graft failure resulting from insufficient flow can be a major issue in current surgical revascularization. Intraoperative flow has been generally accepted as a predictor of patency in arterial and venous bypass grafts [4],[5]; however, the cut-off value remains controversial. Di Giammarco and colleagues reported that graft flow < 15 ml/min was a significant predictor of early graft occlusion of arterial and venous grafts [6] with more than half of all bypass grafts with < 15 ml/min occluding within several months [6]. Tokuda and colleagues suggested cut-off values of 15 ml/min for LCX and 20 ml/min for RCA [7]. In the present study, FI was defined as ≤ 20 ml/min and our definition was not used for detection of technical failure, but to assess the increased risk of graft failure. The value was considered acceptable because FI significantly predicted future NF. Choosing this value added the advantage to this study that graft flow was not biased by cardiopulmonary bypass and cardiac ischemia [8]. When discussing the cut-off value for intraoperative reanastomosis, anticipating flow demand in the grafted region is essential.

With graft capacity, two additional factors influence graft flow. The first is flow demand in the grafted region. Insufficient demand can cause insufficient graft flow and resultant occlusion, even when no competitive flow occurs. In contrast, abundant flow demand, meaning low peripheral vascular resistance or good run-off, is associated with abundant viable myocardium in the grafted region, and is considered beneficial for graft patency. Despite this information, little has been proven regarding flow demand. In this study, we examined the detailed factors regarding flow demand, which are influenced by the size of the revascularized region and myocardial condition. The second factor is native coronary flow. Fractional flow reserve (FFR) is the most reliable current assessment method for the functional significance of the stenosis, compared with conventional visual assessment, especially for moderately stenotic lesions [9],[10]. However, a possible disadvantage is that FFR can be affected by collateral vessels to other vascular regions with chronic total occlusion in multi-vessel disease [11],[12]. We presume that competitive flow can be efficiently avoided by preoperative FFR measurement but graft failure associated with insufficient flow demand will remain.

MLD is another useful alternative when evaluating native coronary stenosis [13]. Fishier and colleagues demonstrated that when MLD was > 1.4 mm, or stenosis was calculated as < 60% by quantitative coronary angiography, FFR was > 0.75 and stenosis was not considered to be functionally significant [14]. In aiming to improve predictive value, we examined MLD in combination with stenosis location, rather than assess MLD alone.

Graft selection for LCX and RCA is a major concern. For LCX, in-situ ITA grafting should be considered first because of improved long-term clinical outcomes [15]. However, using SVG is appropriate in select patients, when failure of ITA is highly likely. For GEA to RCA grafting, Suma and colleagues suggested ≥ 90% stenosis [16], and Kim and colleagues recommended > 80% stenosis and revision of its inflow to the ascending aorta or ITA if graft flow is < 15 ml/min [17]. Glineur and colleagues reported that in-situ GEA was not suitable for MLD > 1.1 mm [13].

The implications of this study are as follows: Irrespective of the relatively short follow-up period, FI was significantly associated with graft failure, and therefore, avoiding FI should be necessary for long-term patency. We found that, even with the same graft material or the same vascular region, the cut-off MLD varied according to flow demand in the grafted region. The stenosis location, which correlates with flow demand, was significantly associated with FI and postoperative graft function. For distal lesions, more severe stenosis with small MLD was necessary to prevent graft failure, because of the small size of the revascularized region. Especially for RCA with distal lesions, the incidence of FI was extremely high, irrespective of graft type and stenosis severity likely because of the limited flow demand. Therefore, grafting indications should be carefully determined according to not only the stenosis severity, but also stenosis location and the viability of that region.

Dion and colleagues have recommended sequential SVG grafting to the distal portion of RCA and LCX [18]. Gao and colleagues also showed that sequential SVG provided a higher patency rate [4] and adjusting the graft length and angle for sequential anastomoses is considered technically easy in off-pump procedures. Consequently, for RCA with a distal lesion, sequential grafting combined with the LCX branch with good run-off may be preferable.

A history of myocardial infarction in the grafted region significantly correlated with FI but left ventricular mass and ejection fraction did not correlate with outcomes in this study. Future studies involving quantified assessment of regional flow demand are needed.

Regarding conduit choice, our results confirmed that SVG was more reliable than arterial grafts when MLD is larger than the cut-off value for arterial grafts and lower than that for SVG. This finding is consistent with the conduit choice for RCA reported by Glineur and colleagues [19]. If MLD is larger than the cut-off value for SVG, deferral of grafting or stent implantation can be considered.

No cut-off value was identified for SVG to LCX with proximal and distal lesions, and SVG and GEA to RCA with distal lesions. We suggest two reasons for this absence of a cut-off MLD. The first is independence from the native coronary flow and the second is the particularly small flow demand in the relevant area, such as with a distal lesion in RCA, as discussed earlier.

The limitations of this study are as follows: First, this study was not prospective or randomized; therefore, we could not perform flow measurements while correcting for physiological conditions, such as heart rate and blood pressure. Second, we selected only those bypass grafts created as the sole bypass graft to the coronary vascular region. This study was performed to determine the impact of flow demand, which is associated with the size of the revascularized region and myocardial condition. Therefore, it was necessary to exclude sequential grafts and patients with two or more bypass grafts in the same vascular region to minimize bias. Despite this, bias regarding the dominancy of three vascular regions could not be eliminated. Third, peripheral vascular resistance and recovery of vasoreactivity commonly decrease postoperatively [20] and these could not be assessed in this study.

## Conclusions

In conclusion, for bypass targets with MLD greater than the cut-off value, a distal lesion, or history of myocardial infarction, in-situ arterial grafts should be applied carefully, especially in RCA and LCX regions. Both competitive flow and insufficient flow demand can cause early graft failure. To prevent IF resulting from insufficient demand, it is essential to assess both flow demand and native vessels. The combination of MLD and stenosis location is efficient in predicting graft functionality and conduit choice. Accurate anticipation of graft flow by quantifying flow demand is future research areas.

## Authors’ original submitted files for images

Below are the links to the authors’ original submitted files for images. Authors’ original file for figure 1 Authors’ original file for figure 2

## Abbreviations

CABG: Coronary artery bypass grafting
FI: Flow insufficiency
GEA: Gastroepiploic artery
ITA: Internal thoracic artery
MLD: Minimal luminal diameter
NF: Not functional
LAD: Left anterior descending artery
LCX: Left circumflex artery
SVG: Saphenous vein graft
