# Supplementary figures and images for: Predictors and prevention of flow insufficiency due to limited flow demand
Source: J Cardiothorac Surg. 2014 Dec 4;9:188. doi: 10.1186/s13019-014-0188-3 (PMC4264538; doi:10.1186/s13019-014-0188-3)

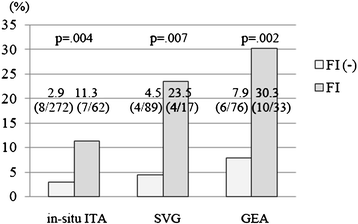

Supplement: Supplementary file 1 — Authors’ original file for figure 1 [file 13019_2014_188_MOESM1_ESM.gif]

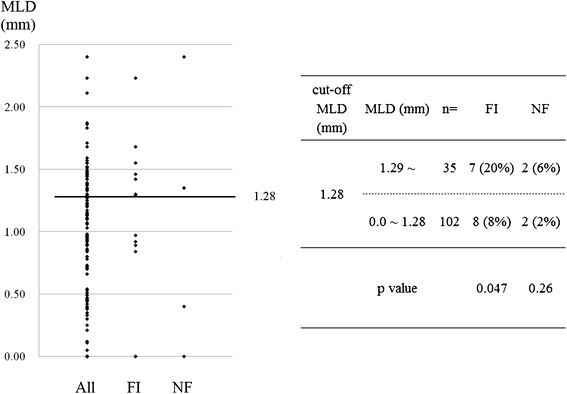

Supplement: Supplementary file 2 — Authors’ original file for figure 2 [file 13019_2014_188_MOESM2_ESM.gif]
